# Metagenome Sequencing of Prokaryotic Microbiota Collected from Rivers in the Upper Amazon Basin

**DOI:** 10.1128/genomeA.01450-16

**Published:** 2017-01-12

**Authors:** Célio Dias Santos-Júnior, Luciano Takeshi Kishi, Danyelle Toyama, Andrea Soares-Costa, Tereza Cristina Souza Oliveira, Fernando Pellon de Miranda, Flávio Henrique-Silva

**Affiliations:** a Department of Genetics and Evolution, Center for Biological and Health Sciences, Federal University of São Carlos, São Carlos, São Paulo, Brazil; b Department of Chemistry, Federal University of Amazonas, Manaus, Amazonas, Brazil; c Centro de Pesquisas e Desenvolvimento Leopoldo Américo Miguez de Mello, Rio de Janeiro, Brazil

## Abstract

Tropical freshwater environments, like rivers, are important reservoirs of microbial life. This study employed metagenomic sequencing to survey prokaryotic microbiota in the Solimões, Purus, and Urucu Rivers of the Amazon Basin in Brazil. We report a rich and diverse microbial community.

## GENOME ANNOUNCEMENT

The Amazon Basin spans eight countries and occupies approximately 40% of the South American continent (1). This basin holds 17% of the planet’s freshwater (2). Moreover, the species richness of its plants and animals is currently estimated to be five times that of North America (3), and more species are being discovered every year (4). However, little is known about its microbiota. Previous studies (1, 5) described the initial microbial landscape of freshwater Amazon rivers being rich in *Actinobacteria*, *Alphaproteobacteria*, *Betaproteobacteria*, *Gammaproteobacteria*, and *Crenarchaeota*. Some marine clades, e.g., *Ilumatobacter*, were also reported (5).

The runoff from rivers in this region account for nearly 15% of the world’s runoff from rivers. There are two well-established seasons (dry and flood) as described in references 6–8. These complex ecosystems work actively in carbon budget processes (9–11).

Because of the presence of noncultivable microorganisms in environmental samples, it is impossible to isolate many of these organisms and difficult to recreate environmental microbiotas in the laboratory. Thus, the most appropriate approach to gain details regarding the community structure is metagenomics. Despite its importance, data on the microbial diversity of the Upper Amazon Basin are very limited. Therefore, this study aimed to form a landscape of the most frequent species in this environment.

We sequenced total DNA extracted from freshwater samples collected from different rivers in the Upper Amazon Basin. These samples were collected in September 2008 (dry season) from the Solimões (03°17′03.86″S, 60°01′23.04″W), Purus (03°41′07.4″S, 61°28′13.91″W), and Urucu (04°08′08.03″S, 63°29′54.69″W) Rivers. Sampling and total DNA extractions were performed as previously described (1). Total DNA was sequenced on an Illumina HiSeq 2500 platform (Illumina, Inc., San Diego, CA); an average of 300,095,181 paired reads of 100 bp per sampling site were obtained. Reads were quality filtered for ambiguities and homopolymers using the next-generation sequencing (NGS) QC Toolkit (12); bases with Phred quality scores <20 were removed.

Taxonomic assignment was made using the MG-RAST server (13) and revealed that 95.4 to 95.8%, 0.3 to 2.2%, and 1.0 to 1.8% of the assigned reads were bacteria, archaea, and viruses, respectively. Major bacterial phyla observed were *Proteobacteria* (abundance percentages of 34.9 to 54.5%), *Actinobacteria* (14.9 to 24.3%), *Cyanobacteria* (1.9 to 24.3%), *Bacteroidetes* (2.0 to 6.6%), *Planctomycetes* (1.8 to 8.2%), and *Firmicutes* (2.5 to 3.2%). Archaea were mainly assigned to *Euryarchaeota* (0.2 to 0.5%) and *Thaumarchaeota* (0 to 1.6%). Alpha diversity was estimated to be between 346 and 620 species. Functional annotations revealed that a considerable portion of reads were associated with respiration (3.4 to 3.7%), photosynthesis (0.2 to 0.8%), and phages, prophages, transposable elements, and plasmids (1.9 to 2.2%). The present metagenomic project holds important insights on the metabolic potential of microbial communities from tropical freshwater rivers.

### Accession number(s).

The sequences obtained in this project have been deposited in the NCBI Short Read Archive under the following accession numbers: SRR1514963 (Solimões River), SRR1515032 (Purus River), and SRR1518285 (Urucu River).
